# Sleep

**Published:** 1878-02

**Authors:** 


### SLEEP.

There is no fact more clearly established in the physiology of man than this, that the brain expends its energies and itself during the hours of wakefulness, and that these are recuperated during sleep; if the recuperation does not equal the expenditure, the brain withers—this is insanity. Thus it is, that in early English history, persons who were condemned to death by being prevented from sleeping, always died raving maniacs; thus it is also, that those who are starved to death become insane; the brain is not nourished, and they cannot sleep. The practical inferences are three :

1st. Those who think most, who do most brain work, require most sleep.

2d. That time "saved" from necessary sleep, is infallibly destructive to mind, body and estate.

3d. Give yourself, your children, your servants, give all who are under you, the fullest amount of sleep they will take, by compelling them to go to bed at some regular, early hour, and to rise in the morning the moment they *awake of themselves;* and within a fortnight nature, with almost the regularity of the rising sun, will unloose the bonds of sleep. the moment enough repose has been secured for the wants of the system This is the only safe and sufficient rule; and as to the question *how much sleep any one requires*, each must be a rule for himself; great Nature will never fail to write it out to the observer, under the regulations just given.

